# White matter diffusion alterations precede symptom onset in autosomal dominant Alzheimer’s disease

**DOI:** 10.1093/brain/awy229

**Published:** 2018-09-25

**Authors:** Miguel Ángel Araque Caballero, Marc Suárez-Calvet, Marco Duering, Nicolai Franzmeier, Tammie Benzinger, Anne M Fagan, Randall J Bateman, Clifford R Jack, Johannes Levin, Martin Dichgans, Mathias Jucker, Celeste Karch, Colin L Masters, John C Morris, Michael Weiner, Martin Rossor, Nick C Fox, Jae-Hong Lee, Stephen Salloway, Adrian Danek, Alison Goate, Igor Yakushev, Jason Hassenstab, Peter R Schofield, Christian Haass, Michael Ewers

**Affiliations:** 1 Institute for Stroke and Dementia Research, Klinikum der Universität München, Ludwig-Maximilians-Universität LMU, Munich, Germany; 2 German Center for Neurodegenerative Diseases (DZNE, Munich), Munich, Germany; 3 Biomedical Center, Biochemistry, Ludwig-Maximilians-Universität München, Munich, Germany; 4 Department of Radiology, Washington University in St Louis, St Louis, MO, USA; 5 Knight Alzheimer’s Disease Research Center, Washington University in St. Louis, St. Louis, MO, USA; 6 Hope Center for Neurological Disorders, Washington University in St. Louis, St. Louis, MO, USA; 7 Department of Radiology, Mayo Clinic, Rochester, MN, USA; 8 Department of Neurology, Ludwig-Maximilians-Universität München, Munich, Germany; 9 Munich Cluster for Systems Neurology (SyNergy), Munich, Germany; 10 Hertie Institute for Clinical Brain Research, Tübingen, Germany and German Center for Neurodegenerative Diseases (DZNE), Tübingen, Germany; 11 Department of Psychiatry, Washington University in St Louis, St Louis, MO, USA; 12 The Florey Institute, The University of Melbourne, Parkville, Victoria, Australia; 13 University of California at San Francisco, San Francisco, CA94143, USA; 14 Dementia Research Centre, University College London, Queen Square, London, UK; 15 Department of Neurology, University of Ulsan College of Medicine, Asan Medical Center, Seoul, Korea; 16 Department of Neurology, Warren Alpert Medical School of Brown University, Providence, Rhode Island, USA; 17 Department of Genetics and Genomic Sciences, Icahn School of Medicine at Mount Sinai, New York, New York, USA; 18 Ronald M. Loeb Center for Alzheimer’s Disease, Department of Neuroscience, Icahn School of Medicine at Mount Sinai, New York, New York, USA; 19 Department of Nuclear Medicine, Technical University of Munich, Munich, Germany; 20 Department of Neurology, Washington University in St. Louis, St. Louis, MO, USA; 21 Neuroscience Research Australia, Barker Street Randwick, Sydney, Australia; 22 School of Medical Sciences, University of New South Wales, Sydney, Australia

**Keywords:** Alzheimer’s disease, autosomal dominant, white matter, diffusion tensor imaging, TREM2

## Abstract

White matter alterations are present in the majority of patients with Alzheimer’s disease type dementia. However, the spatiotemporal pattern of white matter changes preceding dementia symptoms in Alzheimer’s disease remains unclear, largely due to the inherent diagnostic uncertainty in the preclinical phase and increased risk of confounding age-related vascular disease and stroke in late-onset Alzheimer’s disease. In early-onset autosomal-dominantly inherited Alzheimer’s disease, participants are destined to develop dementia, which provides the opportunity to assess brain changes years before the onset of symptoms, and in the absence of ageing-related vascular disease. Here, we assessed mean diffusivity alterations in the white matter in 64 mutation carriers compared to 45 non-carrier family non-carriers. Using tract-based spatial statistics, we mapped the interaction of mutation status by estimated years from symptom onset on mean diffusivity. For major atlas-derived fibre tracts, we determined the earliest time point at which abnormal mean diffusivity changes in the mutation carriers were detectable. Lastly, we assessed the association between mean diffusivity and cerebrospinal fluid biomarkers of amyloid, tau, phosphorylated-tau, and soluble TREM2, i.e. a marker of microglia activity. Results showed a significant interaction of mutations status by estimated years from symptom onset, i.e. a stronger increase of mean diffusivity, within the posterior parietal and medial frontal white matter in mutation carriers compared with non-carriers. The earliest increase of mean diffusivity was observed in the forceps major, forceps minor and long projecting fibres—many connecting default mode network regions—between 5 to 10 years before estimated symptom onset. Higher mean diffusivity in fibre tracts was associated with lower grey matter volume in the tracts’ projection zones. Global mean diffusivity was correlated with lower cerebrospinal fluid levels of amyloid-β_1-42_ but higher levels of tau, phosphorylated-tau and soluble TREM2. Together, these results suggest that regionally selective white matter degeneration occurs years before the estimated symptom onset. Such white matter alterations are associated with primary Alzheimer’s disease pathology and microglia activity in the brain.

## Teaser

Despite the prevalence of white matter alterations in Alzheimer's disease, their occurrence in the presymptomatic phase is poorly understood. Araque-Caballero *et al*. report that diffusivity alterations occur first within the anterior and posterior callosal fibres approximately 10 years before the onset of dementia symptoms in autosomal dominant Alzheimer's disease.

## Introduction

In Alzheimer’s disease, white matter degeneration is a frequent structural brain change apart from grey matter atrophy (Brun and Englund, 1986). Post-mortem studies showed loss of myelin, axonal degeneration and gliosis in the brains from patients with Alzheimer’s disease (Brun and Englund, 1986). Such white matter alterations cannot be fully accounted for as degenerative processes secondary to grey matter damage (Brun and Englund, 1986; Englund, 1998; Agosta *et al.*, 2011), but may stem from vascular amyloid deposition and microvascular damage (Weller *et al.*, 2009). According to the vascular hypothesis, cerebrovascular alterations may contribute to neurodegeneration and white matter damage by reducing the drainage of amyloid and increasing the production of amyloid-β (Zlokovic, 2011). In support of this hypothesis, recent evidence from neuroimaging studies suggests that vascular alterations such as cerebrovascular resistance and hypoperfusion may precede amyloid deposition and are predictive of future disease progression (Iturria-Medina *et al.*, 2016; Yew *et al.*, 2017). Thus, it is possible that vascular and amyloid deposition are synergistically involved in Alzheimer’s disease and contribute to white matter alterations. Higher levels of white matter alterations are associated with FDG-PET decline (Lo *et al.*, 2012; Glodzik *et al.*, 2014; Marnane *et al.*, 2016), higher grey matter atrophy (Bos *et al.*, 2017), reduced functional network integrity (Taylor *et al.*, 2017) and worse cognitive decline (Lo *et al.*, 2012; Marchant *et al.*, 2012; Vemuri *et al.*, 2015). Thus, white matter alterations, possibly stemming from cerebrovascular disease, may add to the effects of amyloid deposition on neurodegeneration and cognition in ageing and Alzheimer’s disease.

A major question is when and where in the brain white matter alterations occur during the course of Alzheimer’s disease. So far, biomarker studies demonstrated the successive development of primary pathologies including amyloid-β and tau (as measured by biofluid and molecular PET markers) followed by alterations of medial temporal and parietal grey matter (as measured by volumetric MRI and FDG-PET) in both sporadic late-onset Alzheimer’s disease (Jack *et al.*, 2012) and genetically-caused Alzheimer’s disease (Bateman *et al.*, 2012). However, the integration of white matter changes within standard biomarker models of the pathological cascade in Alzheimer’s disease remains missing (Jack *et al.*, 2013). The major goal of the current study was to close this research gap and determine the regional patterns of microstructural white matter alterations in different stages of Alzheimer’s disease.

White matter alterations can be detected *in vivo* by diffusion tensor imaging (DTI) (Alexander *et al.*, 2007). Results from DTI showed that fibre tract alterations are widespread in the brain at the stage of mild cognitive impairment and Alzheimer’s disease dementia (Bozzali *et al.*, 2002; Medina *et al.*, 2006; Acosta-Cabronero *et al.*, 2010, 2012; Sexton *et al.*, 2011; Bosch *et al.*, 2012; for reviews see Acosta-Cabronero and Nestor, 2014; Teipel *et al.*, 2015). In particular, major fibre tracts such as the forceps major and forceps minor, inferior fronto-occipital fasciculus as well as the posterior cingulum show white matter alterations in Alzheimer’s disease dementia (Taylor *et al.*, 2001; Teipel *et al.*, 2002; Douaud *et al.*, 2011; Mito *et al.*, 2018). Those fibre tracts connect regions of the default mode network (DMN) and their degeneration may thus contribute to the impairment of the DMN and other functional networks in Alzheimer’s disease (Taylor *et al.*, 2017). Despite these previous findings in symptomatic Alzheimer’s disease participants, white matter changes in the preclinical phase before the occurrence of cognitive impairment have been examined only in a few studies (Gold *et al.*, 2012). In asymptomatic subjects at genetic risk of Alzheimer’s disease (Bendlin *et al.*, 2010; Gold *et al.*, 2010; Cavedo *et al.*, 2017) or with abnormal Alzheimer’s disease biomarker values (Li *et al.*, 2014; Racine *et al.*, 2017), results are mixed including DTI-assessed white matter alterations predominantly in the cingulum, corpus callosum and limbic fibres (Bendlin *et al.*, 2010; Gold *et al.*, 2010; Racine *et al.*, 2017), but also absence of any DTI changes (Li *et al.*, 2014). A difficulty in the interpretation of these previous findings is the uncertain future development of dementia symptoms at the presumed preclinical stage of Alzheimer’s disease. In contrast, in autosomal-dominant Alzheimer’s disease mutation carriers will all develop dementia and the future onset of dementia symptoms can be more precisely estimated (Ryman *et al.*, 2014). Furthermore, because of the younger age at onset of autosomal-dominant Alzheimer’s disease, there is less likelihood of confounding vascular disease. However, it should be cautioned that results from autosomal-dominant Alzheimer’s disease do not necessarily generalize to sporadic late-onset Alzheimer’s disease. Age-related vascular changes may influence disease progression in late-onset Alzheimer’s disease, including the development of white matter alterations (Iturria-Medina *et al.*, 2016), which is less likely to be captured in autosomal dominant Alzheimer’s disease where dementia symptoms typically emerge between 30 and 50 years of age. Nevertheless, neuroimaging and biomarker studies show consistencies in the sequential onset of amyloid deposition, tau, FDG-PET hypometabolism, grey matter atrophy, and clinical presentation between late-onset and autosomal-dominant Alzheimer’s disease (Bateman *et al.*, 2012; Benzinger *et al.*, 2013; Young *et al.*, 2014; Gordon *et al.*, 2018; Oxtoby *et al.*, 2018), suggesting substantial commonalities between both sporadic late-onset and genetically caused Alzheimer’s disease. A few DTI studies on white matter alterations in autosomal-dominant Alzheimer’s disease have been previously conducted (Ringman *et al.*, 2007; Ryan *et al.*, 2013; Parra *et al.*, 2015; Sanchez-Valle *et al.*, 2016). However, these studies either lacked sufficient statistical power considering the relatively low sample sizes (*n = *8 to 18 of asymptomatic carriers) to detect subtle pre-symptomatic phase or did not estimate years from symptom onset (EYO) (Ringman *et al.*, 2007; Ryan *et al.*, 2013; Parra *et al.*, 2015), which reduced the sensitivity to detect DTI changes that occur in close proximity to the onset of dementia symptoms.

In the current study, our primary aim was to map DTI alterations as a function of EYO and to place the time course of white matter alterations within the temporal biomarker model of Alzheimer’s disease. We controlled for the influence of white matter hyperintensities, i.e. macrostructural white matter changes that can significantly alter DTI measures such as mean diffusivity (Medina *et al.*, 2006). A secondary aim was to test whether CSF-based biomarker alterations of Alzheimer’s disease pathology and microglia activity are related to mean diffusivity alterations. This aim was motivated by recent findings of a close relationship between tau pathology and white matter diffusion alterations (Sydykova *et al.*, 2007; Kantarci *et al.*, 2017; Jacobs *et al.*, 2018), as well as alterations of CSF levels of TREM2, a marker of microglia activity in Alzheimer’s disease (Kleinberger *et al.*, 2014; Suarez-Calvet *et al.*, 2016*a*, *b*). For all analyses, we used mean diffusivity as our primary DTI measure in autosomal-dominant Alzheimer’s disease, since previous studies found mean diffusivity to be the most sensitive DTI marker to detect fibre tract alterations (Clerx *et al.*, 2012; Nowrangi *et al.*, 2013).

## Materials and methods

### Study design

#### Participants

We included 153 subjects from the Dominantly Inherited Alzheimer Network (DIAN, data freeze 9). Participants enrolled in DIAN were examined on the basis of a comprehensive battery of neuropsychological testing, neuroimaging assessments and biomarker (CSF) measurements. EYO was determined based on the age of onset of the parent from whom they inherited the mutation (Bateman *et al.*, 2012). The current study is a subset of the 218 participants in whom we reported previously CSF soluble TREM2 changes (Suarez-Calvet *et al.*, 2016*a*). For the current study, additional inclusion criteria were: availability of DTI scan (*n = *42 participants excluded), and acquisition with 64 diffusion directions (*n = *23 participants excluded). Quality control applied to the remaining DTI scans resulted in the exclusion of an additional 37 participants because of only partial coverage of the brain due to a restricted field of view. Excluded were also cases with abnormalities that prevented a valid DTI analysis, including frontal DTI signal loss (*n = *1), intracerebral haemorrhage (*n = *3), severe grey matter atrophy (*n = *1), signs of cerebral amyloid angiopathy (*n = *1, number of microbleeds = 100), or an extremely large white matter hyperintensity volume [*n = *1, 25 × the interquartile range (IQR) above the median within the mutation carriers, Tukey criterion of outlier defined as 1.5 × IQR]. This final sample of *n = *109 participants comprised 64 carriers and 45 non-carriers (Table 1). Mutations occurred within the *PSEN1* (*n = *47), *PSEN2* (*n = *8), and *APP* (*n = *9) genes. Compared to the carriers who were excluded, those carriers included were slightly younger [mean (standard deviation, SD) = 38.3 (11.3) versus 41.5 (10.0) years, *P* = 0.04] and had a lower EYO [mean (SD) = −9.1 (11.2) versus −4.6 (9.9) EYO, *P = *0.02]. No differences in gender distribution, *APOE* genotype or years of education were present between the carrier groups, nor were any differences in those variables detected between included and excluded non-carriers. The proportion of carriers versus non-carriers was similar between the final sample and the excluded participants (57.2% versus 61.9%, *P = *0.6).

**Table 1:** Subject characteristics

Group	Non-carriers (*n* = 45)	Carriers (*n* = 64)
Gender, male/female (%)	24 (53) / 21 (47)	28 (44) / 36 (56)
Age, years, mean ± SD	38.8 ± 10.6	38.0 ± 11.2
EYO, years, mean ± SD	–5.8 ± 11.3	–8.4 ± 11.6
Education, years (mean ± SD)	15.1 ± 2.0	14.0 ± 3.6*
*APOE* ɛ4, NC/C (%)	30 (67) / 15 (33)	50 (78) / 14 (22)
Symptomatic, A/S (%)	44 (98) / 1 (2)	32 (52) / 31 (48)^a^

A = asymptomatic; C = carrier; NC = non-carrier; S = symptomatic.

*P < 0.05 (two-sample *t*-test).

^a^One carrier had no clinical dementia rating available.

#### MRI scan acquisition

Whole-brain T_1_-weighted MPRAGE scans (repetition time = 2300 ms, echo time = 2.95 ms, flip angle = 9°) with 1.1 mm × 1.1 mm × 1.2 mm voxel size were acquired.

The DTI scans consisted of whole-brain T_2_*-echo planar images (repetition time = 11 000 ms, echo time = 87 ms). The acquisition was performed axially with an anterior-to-posterior phase-encoding direction. One reference volume (b = 0 s/mm^2^) and 64 diffusion volumes (b = 1000 s/mm^2^) with uniformly distributed diffusion directions were acquired with isotropic 2.5 mm voxel size. That standardized DTI protocol was implemented exclusively on 3 T MRI scanners with a 20-channel head coil from the manufacturer Siemens at 10 centres in DIAN. Sites were qualified by phantom and human volunteer scans, and quality control of scans by a central laboratory (C.J., Mayo Clinic) was implemented to reduce between-centre variability that may arise from varying filters for image reconstruction, gradient strength and other magnetic resonance features set by vendor-specific magnetic resonance protocols. T_2_-FLAIR scans were acquired axially (repetition time = 9000 ms, echo time = 91 ms, flip angle = 150°) with 0.9 mm × 0.9 mm × 5.0 mm voxel size.

#### Preprocessing of MRI scans

The DTI scans were corrected for motion, eddy-current and susceptibility artefacts using a pipeline implemented in ExploreDTI (http://www.ExploreDTI.com). Briefly, for motion correction, rigid-body rotations using the b = 0 scan as a reference image were applied to both the b = 1000 s/mm^2^ volumes and B-matrix of diffusion directions (Leemans and Jones, 2009). Eddy-current correction was performed by affine transformation of the b = 1000 s/mm^2^ volumes to the B0 scan. Correction for susceptibility distortion was performed by non-linear registration of the diffusion-weighted and B0 scans to the subject’s brain-extracted T_1_, using the brain masks as priors and allowing only for deformations in the phase-encoding direction (Irfanoglu *et al.*, 2012). After all corrections were performed, the parameters of the diffusion tensor were estimated via weighted least-squares to generate maps of mean diffusivity. Mean diffusivity was our main DTI-derived quantity of interest, but for exploratory analyses we also generated maps of other commonly used DTI indices including fractional anisotropy, radial diffusivity and axial diffusivity previously found to be altered in Alzheimer’s disease (for review see Acosta-Cabronero and Nestor, 2014). Subsequently, each subject’s maps of the different DTI indices were projected onto the standard FMRIB-58 fractional anisotropy template in MNI space and skeletonized. This was conducted via the Tract-Based Spatial Statistics (TBSS) toolbox of FSL (https://fsl.fmrib.ox.ac.uk/fsl/fslwiki), using the implemented spatial normalization routing and standard parameters (Smith *et al.*, 2006). We applied a white matter skeleton mask from the TBSS toolbox, which was modified such that the fornix and other regions prone to partial-volume effects were excluded, as established previously (Baykara *et al.*, 2016).

#### White matter hyperintensity segmentation

White matter hyperintensities (WMHs) were identified based on FLAIR scans, using a semi-automated in-house method described previously (Taylor *et al.*, 2017). For further details see the Supplementary material.

#### Detection of microbleeds and superficial cortical siderosis

To assess the influence of cerebral amyloid angiopathy-related small vessel disease on white matter changes, we rated microbleeds and superficial cortical siderosis. Based on T_2_*-weighted gradient echo images, which were available in a subset of subjects (*n = *71), microbleeds and superficial cortical siderosis were assessed via visual inspection by an expert rater (M.D.). Microbleeds were rated according to the STRIVE consensus criteria (Wardlaw *et al.*, 2013).

#### Determination of grey matter volume in projection zones of fibre tracts

In the first step, those fibre tracts that contained significant alterations in autosomal-dominant Alzheimer’s disease were determined. To this end, the Johns Hopkins University whole-brain tractography atlas (in MNI space) (Wakana *et al.*, 2007), was superimposed onto the TBSS-derived statistical map of the EYO × mutation interaction effect (see also ‘Statistical analyses’ section below). Some tracts showed no or only a small number of significant voxels (0.8–1.6% of the total voxel-number per tract) and were excluded from further analysis. This resulted in a set of 8 of 11 fibre tracts. In addition, we *a priori* included the hippocampal cingulum bundle fibre tract on the basis of previous publications showing early alterations of this tract in Alzheimer’s disease (Zhang *et al.*, 2009; Jacobs *et al.*, 2018).

Next, we determined the grey matter projection zones of those selected fibre tracts, using an in-house written MATLAB script. A detailed description can be found in the Supplementary material. Briefly, to reconstruct the streamlines of the fibre tracts, whole-brain tractography based on the Johns Hopkins University DTI template was performed. Inclusion and exclusion regions of interest were placed at anatomical landmarks according to a previously established protocol, in order to extract the fibre tracts of the atlas (Wakana *et al.*, 2007). To determine the projection of the fibres into grey matter, the direction of fibre projection was estimated based on a series of tangents estimated at regularly spaced points within the terminal section of each fibre (Supplementary Fig. 1). Based on the thus estimated direction of the terminal section of a fibre, the fibre was projected into the grey matter mask that was superimposed onto the DTI image in MNI space. The grey matter voxels surrounding the projected fibre defined the grey matter projections zone. The grey matter projection zone masks were superimposed onto each subject’s spatially normalized grey matter image (in MNI space) for extraction of the grey matter volume for each tract and subject.

#### Global white matter changes

In addition to regional mean diffusivity changes, we analysed global mean diffusivity changes, using the DTI-based biomarker ‘peak-width of skeletonized mean diffusivity’ (PSMD), which was recently developed and cross-validated (Baykara *et al.*, 2016). Specifically, in the first step, the intensity distribution of mean diffusivity values within the TBSS-derived white matter skeleton was plotted. Subsequently, the PSMD was computed as the difference between the 5th and 95th percentile of the skeletonized mean diffusivity histogram (within the custom white matter mask). A higher PSMD value indicates a higher dispersion of mean diffusivity values, indicative of local mean diffusivity alterations within the white matter.

#### CSF biomarker assessment

Amyloid-β_1-42_, total tau and hyperphosphorylated tau (P-tau_181_) were analysed by multiplexed Luminex-based immunoassay (INNO-BIA AlzBio3, Fujirebio). CSF soluble TREM2 was measured by an ELISA previously established based on the MSD Platform (Kleinberger *et al.*, 2014; Suarez-Calvet *et al.*, 2016*a*).

#### MRI and amyloid PET markers

For precuneus Pittsburgh compound B (PIB)-PET, we applied a FreeSurfer-based extraction of the binding potential, that was partial-volume corrected based on a regional spread-function approach (Rousset *et al.*, 1998). Of 21 carriers and 24 non-carriers in whom PIB-PET was available, 16 carriers were amyloid positive (mean cortical binding potential > 0.18) (Mintun *et al.*, 2006; Gordon *et al.*, 2016) and none of the non-carriers.

For hippocampus volume, FreeSurfer-based segmentation was used. Correction for total intracranial volume was done using a regression approach as previously described (Jack *et al.*, 1989; Benzinger *et al.*, 2013).

### Statistical analyses

The mean diffusivity values were the primary measure of white matter integrity. Other commonly used DTI indices include fractional anisotropy, radial diffusivity and axial diffusivity, which were included as secondary measures for exploratory reasons.

#### Voxel-wise analysis using TBSS

For the analysis of differences in mean diffusivity between carriers and non-carriers during the course of autosomal-dominant Alzheimer’s disease, we tested linear regression models with mean diffusivity as the dependent variable and the interaction EYO × mutation status, EYO, mutation status, sex and years of education as predictors. Statistical significance was determined through non-parametric tests (500 permutations), as implemented in the toolbox Randomise of FSL (Winkler *et al.*, 2014). Threshold-free cluster enhancement (TFCE) with a significance level of *P = *0.05 (family-wise error corrected) was applied (Smith and Nichols, 2009). Since the hippocampus cingulum bundle is only a thin band of white matter, and thus a cluster-based significance threshold is less sensitive, we relaxed the significance criterion to *P < *0.01 (voxel-based uncorrected) for that tract. The same analyses were run for fractional anisotropy, radial diffusivity and axial diffusivity as dependent variables. To determine common spatial patterns of changes for mean diffusivity, fractional anisotropy, radial diffusivity and axial diffusivity, we binarized each of the TBSS results at the significant threshold level and computed the intersection of the thresholded statistical maps. In addition, we computed voxel-level group differences in mean diffusivity between symptomatic carriers (clinical dementia rating > 0), asymptomatic carriers (clinical dementia rating = 0) and non-carriers, using the same covariates as for the previous analyses.

Next, we illustrated the TBSS-derived interaction effects on mean diffusivity. Since this is not possible for each voxel, we generated interaction plots for each major fibre tract in order to cover regional variations. Specifically, we calculated the average mean diffusivity across all voxels showing significant interaction effects for each fibre tract region of interest. To this end, the Johns Hopkins University fibre tract atlas (Hua *et al.*, 2008) in MNI space was superimposed onto the thresholded TBSS map and the average mean diffusivity within the masks’ intersection was computed.

#### PSMD marker of global white matter change

Lastly, we assessed the interaction effect EYO × mutation status on global mean diffusivity changes using the previously proposed PSMD, a marker of white matter changes. Statistical analyses on PSMD were done in an analogous fashion as for the voxel-based mean diffusivity map.

#### Assessing the influence of white matter hyperintensity on mean diffusivity alterations

To map the spatial distribution of WMH in carriers and non-carriers, we computed the voxel-wise occurrence of WMH as the number of participants exhibiting a WMH in that voxel was divided by the total number of participants in the group (see Supplementary material for further details).

#### Temporal ordering of mean diffusivity changes

To assess how early abnormal mean diffusivity changes occur, we studied the trajectories of mean diffusivity across EYO for each fibre tract, using the same statistical method described previously (Suarez-Calvet *et al.*, 2016*a*). Specifically, we used the average mean diffusivity values for each fibre tract region of interest described above (see ‘Voxel-wise analysis using TBSS’ section above) as the dependent variable to estimate the coefficient for the interaction effect (EYO × mutation status) for each tract. Using robust linear regression analysis with M-estimation (implemented in the R-package MASS) (Venables and Ripley, 2002), we tested predictors including mutation status, EYO, EYO × mutation status, sex and years of education. Prior to the regression analyses, outliers were removed separately in carriers and non-carriers, using the Tukey criterion: average mean diffusivity > 1.5 × IQR > median. At most, five data points (i.e. participants) were removed for any given tract. Based on the thus estimated regression coefficients, we computed point estimates of tract-based average mean diffusivity values and PSMD for each group and EYO (ranging from EYO = −25 to EYO = +10, in 5-EYO steps). For each EYO value we computed 95% confidence intervals (CI) of the difference in the point estimates between carriers and non-carriers at each EYO step. The group differences in mean diffusivity and PSMD were considered statistically significant at a particular EYO if the 95% CI of the group difference did not include zero.

#### Mean diffusivity changes within the biomarker cascade model

We modelled changes in PSMD and forceps major, which showed the earliest changes in mean diffusivity, as a function of EYO. In addition, standard biomarkers included (i) PIB-PET within the precuneus and CSF amyloid-β_1-42_ as biomarkers of amyloid deposition; (ii) CSF total tau as a biomarker of neurodegeneration; (iii) hippocampal volume as a marker of grey matter atrophy (Benzinger *et al.*, 2013; Fagan *et al.*, 2014); and (iv) CSF-soluble TREM2 as marker of microglial activation (Suarez-Calvet *et al.*, 2016*a*). The trajectories for each measure were modelled according to the same statistical methods as described previously (Bateman *et al.*, 2012; Suarez-Calvet *et al.*, 2016*a*). Specifically, starting with linear terms, we successively added higher order terms to increase the overall model fit, using the Akaike information criterion as a penalty function and stopping criterion. For the estimation of the biomarker curves, we applied the thus estimated regression equations, rather than locally weighted scatter plot smoothing (LOESS) (Bateman *et al.*, 2012), to obtain an exact illustration of the estimated regression coefficients. This approach resulted in more linear biomarker curves compared to those reported previously (Bateman *et al.*, 2012).

#### Relationship between mean diffusivity and core autosomal-dominant Alzheimer’s disease biomarkers

We computed robust linear regressions with PSMD as the main outcome and each of the three CSF biomarkers (amyloid-β_1-42_, total tau, P-tau_181_ and soluble TREM2) as well as PIB-PET uptake in the precuneus as the main predictors within the carrier group.

In addition, we computed robust linear regressions with tract-specific mean diffusivity as main predictor and grey matter volume at the projection zone of the tract as main outcome variable, to assess associations between white matter alterations and changes in the grey matter. All analyses included sex and education as covariates.

### Data availability

Both raw and processed data that support the findings of the current study will be made available upon request to the corresponding author and the DIAN committee in order to ensure that the privacy of the DIAN participants is protected.

## Results

### Subject characteristics

Subject characteristics are presented in Table 1. Carriers were less educated than non-carriers [*t*(93.8) = −2.03, *P = *0.045], but the groups did not differ in terms of age, sex distribution and *APOE* ɛ4 genotype distribution.

### Mapping group differences in mean diffusivity across estimated years from symptom onset

**Figure 1:**
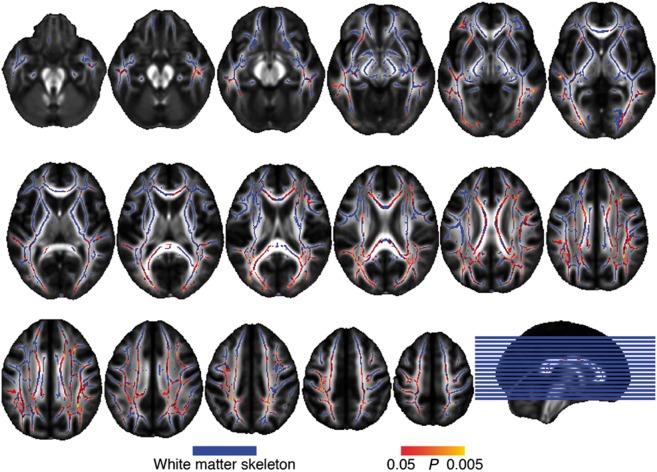
**Abnormal mean diffusivity changes as a function of EYO.** *P*-value map of the statistically significant voxel-wise interactions of EYO × mutation status on mean diffusivity (red-yellow) superimposed on the white matter skeleton (blue, axial view), FWE-corrected at *P = *0.05.

**Figure 2:**
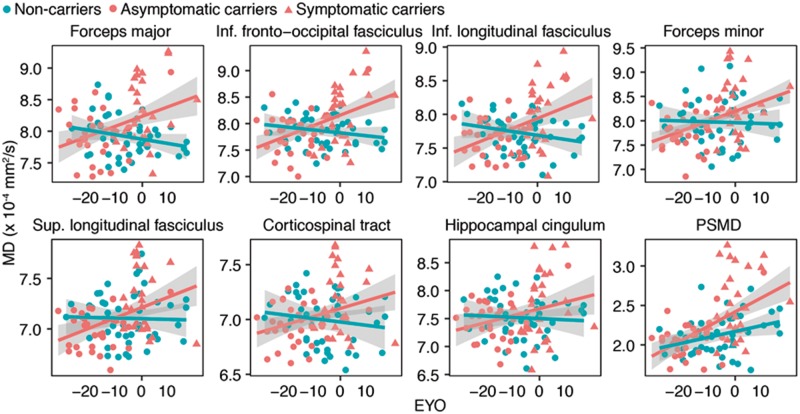
**Tract-specific mean diffusivity changes as a function of EYO.** Regression plots for the interaction EYO × mutation status on mean diffusivity are shown for each fibre tract and the PSMD value. The regression lines including the 95% CIs (shaded bands) for mutation carriers (red) and non-carriers (blue) as a function of EYO are displayed.

We found significant interactions of EYO by mutation status on mean diffusivity predominantly in posterior parietal and medial frontal regions of the white matter (Fig. 1, peak coordinates in Table 2), indicating a faster increase of mean diffusivity in carriers compared to non-carriers. No interactions in the opposite direction were observed. For global white matter differences in mean diffusivity, assessed by the PSMD biomarker, the interaction effect EYO × mutation status was B(standard error, SE) = 1.7(0.6) × 10^−6^, *P < *0.001, indicating a faster progression in PSMD with EYO for carriers. Regression plots of the TBSS-assessed interaction effect EYO × mutation status on mean diffusivity are illustrated at the fibre tract level in Fig. 2. These results on mean diffusivity were largely consistent with those found for alternate DTI indices including fractional anisotropy, radial diffusivity and axial diffusivity (Supplementary material, Supplementary Figs 3–6 and Supplementary Tables 3–5).

**Table 2:** Fibre tract and MNI coordinates of peak T-statistic values of the interaction of EYO × mutation status on mean diffusivity

Cluster	Tract	Reference	*x*	*y*	*z*	T-value
1	Forceps major	Occipital lobe	−24	−79	1	4.73
2	Superior longitudinal fasciculus R	Frontal lobe	30	16	41	4.47
3	Inferior longitudinal fasciculus R	Temporal lobe	43	−23	−16	3.81
4	Superior longitudinal fasciculus L	Temporal lobe	−50	−36	−9	3.69
5	Forceps minor	Frontal lobe	21	24	33	2.99
6	Inferior fronto-occipital fasciculus L	Frontal lobe	−22	22	−5	3.23
7	Superior longitudinal fasciculus L	Caudate	−27	0	26	3.61
8	Superior longitudinal fasciculus L	Temporal lobe	−55	−33	−10	3.39

L = left; R = right. The MNI coordinates are in millimetres.

### Mean diffusivity alterations in symptomatic and non-symptomatic carrier groups

The symptomatic carrier group (clinical dementia rating > 0, *n = *31) showed increased mean diffusivity compared to non-carriers (*n = *32, after matching the EYO range) primarily in regions of the posterior white matter, similar to the TBSS analysis of the interaction EYO × mutation status (Supplementary Fig. 2A and Supplementary Table 1). No group differences in the opposite direction were observed. The results of the regression analysis of the interaction EYO × mutation status within the asymptomatic carrier group and all non-carriers was not significant, probably due to a lack power of this smaller sample. When compared with asymptomatic carriers (clinical dementia rating = 0), symptomatic carriers showed increased mean diffusivity in predominantly parietal regions (Supplementary Fig. 2B and Supplementary Table 2).

### Influence of white matter hyperintensity on mean diffusivity changes

Probability mapping of WMHs showed that WMHs were mostly distributed around the ventricles and in posterior parts of the white matter, which was more pronounced in the carriers compared to the non-carriers (Supplementary Fig. 7). To assess whether the occurrence of WMHs account for the observed alterations in mean diffusivity, we tested the interaction EYO × mutation status on the mean mean diffusivity values for each fibre tract, where the mean diffusivity was averaged either within the WMH or the normal-appearing part of a tract. For each tract, the regression analyses yielded virtually the same interaction effect of EYO × mutation status on mean diffusivity regardless of whether mean diffusivity values were averaged within or outside WMH areas of a particular tract (Supplementary Fig. 8). This indicates that the occurrence of mean diffusivity changes found in carriers were not exclusively dependent on the presence of WMHs.

In addition to WMHs, microbleeds and cortical siderosis was assessed. Only three subjects showed any microbleeds (*n = *1, 4 or 100 in the cortex, respectively). Cortical siderosis was detected in the subject who had one microbleed. Note that the subjects with one and 100 microbleeds had been excluded from all DTI analyses due to intracerebral haemorrhage (in the case with one microbleed) and suspected cerebral amyloid angiopathy (in the case of 100 microbleeds). Thus, microbleeds were unlikely to have any influence on the current analysis.

### Temporal ordering of tract-based mean diffusivity differences

**Figure 3:**
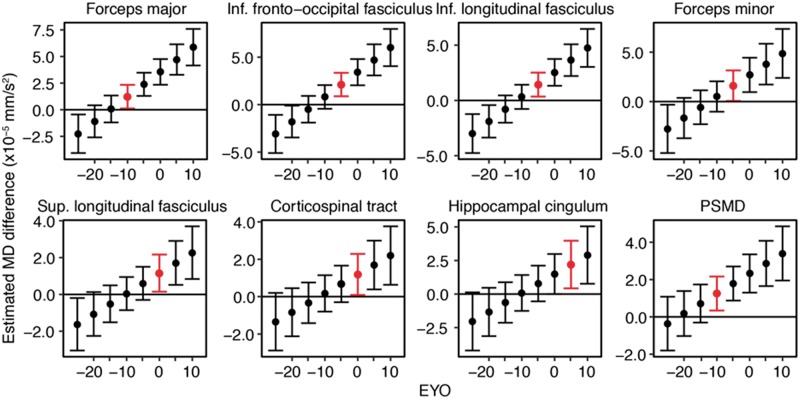
**Time of first detectable changes in mean diffusivity.** Ninety-five per cent confidence intervals of the estimated mean diffusivity and PSMD differences between mutation carriers and non-carriers at each EYO are shown. Red indicates the earliest time point of abnormal mean diffusivity values for a particular tract.

At the tract level, mean diffusivity increases became significant first at EYO = (−10, −5) for the forceps major and forceps minor, respectively (Fig. 3). Subsequently, mean diffusivity differences occurred at EYO = (−5, 0). The inferior fronto-occipital fasciculus, the anterior thalamic radiation and the inferior and superior longitudinal fasciculi. The global mean diffusivity marker PSMD became abnormal in the interval EYO = (−10, −5).

**Figure 4:**
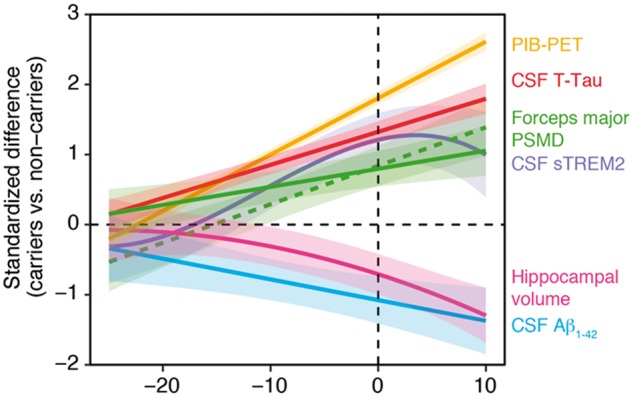
**Trajectories of white matter alterations and biomarkers across EYO.** Trajectories of alterations in mean diffusivity in the forceps major, and PSMD (green) along with those of core Alzheimer’s disease biomarkers and CSF soluble TREM2.

Figure 4 shows the estimated trajectories of changes in the forceps major, PSMD, and standard biomarkers of Alzheimer’s disease. Alterations of mean diffusivity in the forceps major and the PSMD became significant at EYO = (–10, –5). Thus, changes in mean diffusivity appeared after changes in amyloid deposition [CSF amyloid-β_1-42_: EYO = (−15, −10), PIB-PET: EYO = (20, −15)], and tau [CSF total tau: EYO = (−20, −15)]. The white matter changes were concurrent with changes in microglial activation [CSF soluble TREM2: EYO = (−10, −5)] and preceded changes in grey matter atrophy [hippocampal volume: EYO = (−5, 0)].

### Relationship between mean diffusivity and biomarkers of Alzheimer’s disease pathology and microglial activation

To reduce the number of tests, we assessed the relationship between biomarkers for Alzheimer’s disease and mean diffusivity for the global marker PSMD.

**Figure 5:**
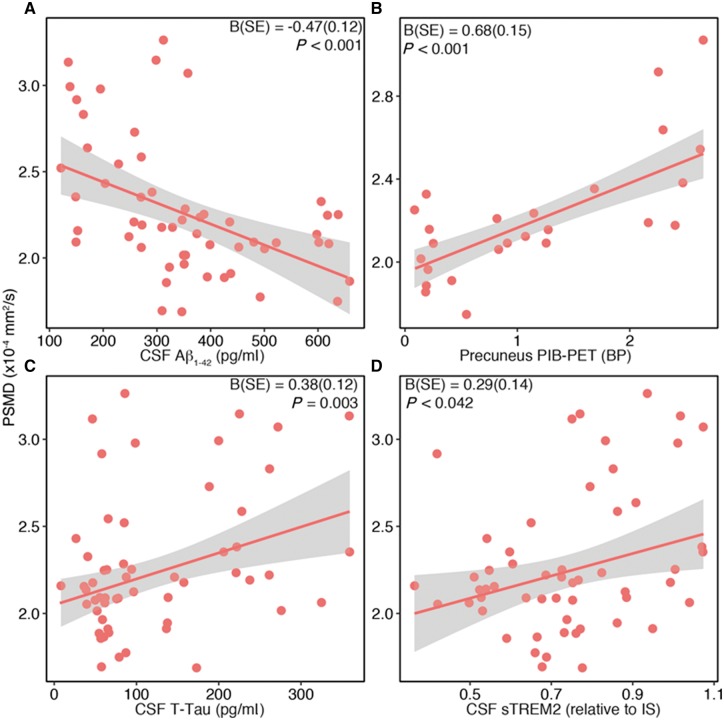
**Global white matter alteration associated with Alzheimer’s disease biomarkers and CSF soluble TREM2.** Regression plots for PSMD versus CSF amyloid-β_1-42_ (**A**), precuneus PIB-PET (**B**), CSF total tau (**C**) and CSF-soluble TREM2 (**D**) in the carrier group. The shaded areas correspond to the 95% CIs of the regression lines. BP = binding potential; IS = internal standard.

Higher PSMD was associated with lower CSF amyloid-β_1-42_ [B(SE) = −0.47(0.12), *P < *0.001; Fig. 5A] and increased PIB-PET binding potential [B(SE) = 0.68(0.15), *P < *0.001; Fig. 5B]. In addition, higher PSMD was associated with higher levels of CSF total tau [B(SE) = 0.38(0.12), *P = *0.003; Fig. 5C], P-tau [B(SE) = 0.41 (0.12), *P < *0.001], and higher levels of the microglia biomarker CSF soluble TREM2, [B(SE) = 0.29(0.14), *P = *0.042; Fig. 5D]. For P-tau_181_ the results were almost indistinguishable from those for total tau due to the high correlation between the two biomarkers (plot not shown).

**Figure 6:**
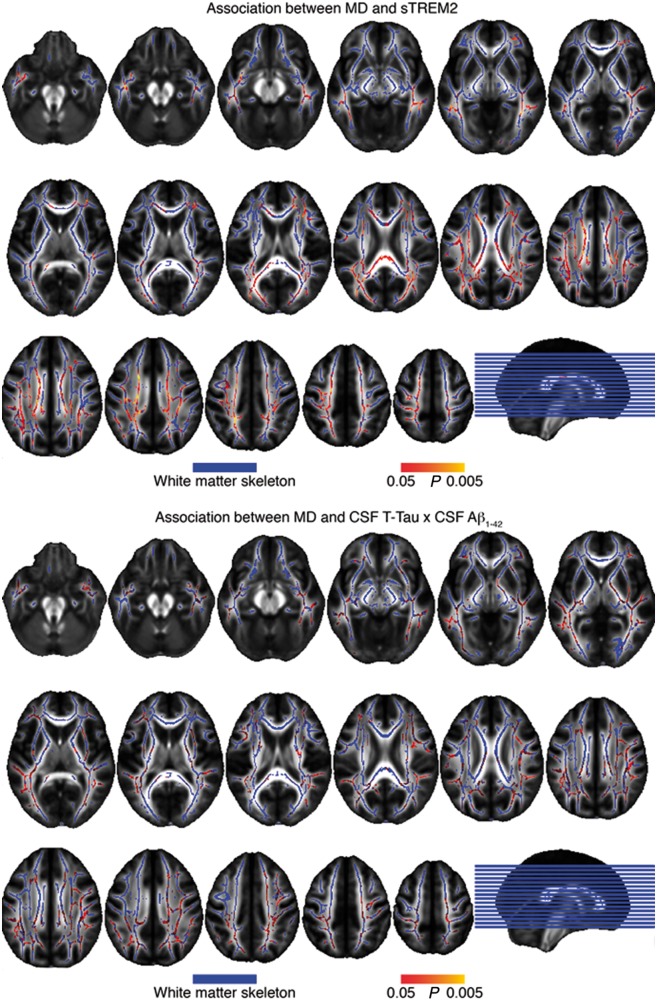
**Association between CSF markers and regional mean diffusivity in the carriers.** *Top*: Map of *P*-values for the association between higher CSF soluble TREM2 and higher mean diffusivity (red–yellow) superimposed on the white matter skeleton (blue), FWE-corrected at *P = *0.05. *Bottom*: Map of *P*-values for the interaction effect of CSF amyloid-β_1-42_ by total tau on mean diffusivity. MD = mean diffusivity.

To test the region-specific association between CSF biomarkers and mean diffusivity, we first conducted a voxel-based regression of mean diffusivity onto TREM2 in the mutation carriers. We found significant associations between mean diffusivity and CSF soluble TREM2 that were predominantly located in temporal and parietal regions of the white matter, including fibre tracts such as the forceps major and minor among other tracts (Fig. 6A, peak coordinates in Supplementary Table 6). Since recent studies suggested that tau is associated with neurodegeneration and white matter diffusivity particularly at high levels of amyloid deposition (Desikan *et al.*, 2011; Jacobs *et al.*, 2018), we tested the interaction between CSF amyloid-β_1-42_ × total tau on mean diffusivity. Results are shown in Fig. 6B (peak coordinates in Supplementary Table 7).

### Relationship between fibre-tract specific mean diffusivity and grey matter volume in the tracts’ projection zones

Fibre tract degeneration has been associated with reduced grey matter volume in connected brain regions (Duering *et al.*, 2012). Here, we found that higher tract-specific mean diffusivity was associated with lower grey matter volume in the projection zones of all tracts except the hippocampal cingulum bundle (for each tract FDR-corrected *P*-value < 0.01; Supplementary Fig. 9).

## Discussion

The major finding of the current study was the region-specific increase in mean diffusivity that emerged about 10 years before the estimated onset of dementia symptoms. These fibre tract alterations were not restricted to WMH areas, suggesting that subtle changes in the normal-appearing white matter were already present years before the onset of dementia symptoms. Higher mean diffusivity was associated with lower CSF amyloid-β_1-42_, higher CSF tau and CSF soluble TREM2, suggesting that early white matter alterations were associated with primary Alzheimer’s disease pathology and microglia activity.

Our current findings provide novel insights to clarify the question of whether white matter alterations precede the onset of dementia symptoms and if so which fibre tracts are affected first in the course of Alzheimer’s disease (Acosta-Cabronero and Nestor, 2014; Teipel *et al.*, 2015). Our results revealed an abnormal increase in mean diffusivity in the forceps major (which projects into the posterior parietal and occipital cortex) a decade before the onset of dementia symptoms, with the forceps minor (which projects into the medial and lateral frontal cortex) and long-ranging projection fibres such as the inferior fronto-occipital fasciculus all showing abnormal mean diffusivity several years before the expected onset of dementia symptoms. Anterior and posterior callosal fibres and fibres such as the inferior-fronto-occipital fasciculus provide major connections within the anterior and posterior components of the default mode network regions (van den Heuvel *et al.*, 2009), a functional network showing reduced connectivity in early-stage Alzheimer’s disease (Sorg *et al.*, 2007; Chhatwal *et al.*, 2013). Although any correlational data preclude a causative conclusion, our findings support the possibility that callosal white matter degeneration contribute to functional disruption of functional networks such as the default mode network (Chhatwal *et al.*, 2013; Mueller and Weiner, 2017; Taylor *et al.*, 2017). The hippocampal cingulum bundle showed alterations only after the expected onset of symptom. However, the cingulum bundle is only a thin white matter tract, which may render a region of interest approach less sensitive to detect diffusion alterations in that tract. It is important to note that current findings on DTI assessed white matter differences show where mean diffusivity alterations can be first detected, but not necessarily where they first occur. The sensitivity to detect white matter alterations via DTI may differ between fibre tracts. In addition to the regional change, we found abnormally increased levels of the PSMD marker of global white matter changes years before estimated symptom onset early. The PSMD is fully automated index is sensitive to regionally heterogeneous abnormal mean diffusivity values (Baykara *et al.*, 2016). The current findings suggest that PSMD was sensitive to the earliest white matter changes observed in the callosal fibres, and may thus be a candidate marker for early white matter changes such as occurring in Alzheimer’s disease.

The current results are consistent with previous studies on presumed preclinical Alzheimer’s disease, reporting decreased fibre tract integrity within the posterior parietal white matter and medial temporal white matter to be associated with imminent conversion from asymptomatic cognitive status to mild cognitive impairment within 2 years (Zhuang *et al.*, 2012) or with the presence of both abnormal levels of CSF amyloid and neuroinjury markers (FDG-PET hypometabolism or CSF tau) (Bendlin *et al.*, 2012; Kantarci *et al.*, 2014). In a similar vein, a combined ante-mortem DTI and post-mortem histochemical analysis in brains from cognitively normal participants found amyloid and tau pathology to be associated with ante-mortem DTI alterations selectively in the medial temporal and parietal white matter (Kantarci *et al.*, 2017). In contrast, increased amyloid deposition alone was not found to be associated with DTI changes at the preclinical stage. The current findings on the estimated trajectories of DTI and biomarkers of Alzheimer’s disease pathology extend these previous results suggesting that subsequent to the elevation of amyloid and tau pathology, alterations in frontal and parietal white matter regions start to develop a decade before dementia onset.

WMHs are considered an MRI detectable marker of white matter changes due to microvascular pathology, and are increased in autosomal-dominant Alzheimer’s disease (Lee *et al.*, 2016). Our DTI-based results demonstrated an increase in mean diffusivity with disease progression both inside and outside areas of WMH. Importantly, this finding does not dispute the possible contribution of amyloid-β-related small vessel changes to the observed white matter alterations. WMHs manifest as regions of increased diffusivity in DTI and likely reflect the tip of the iceberg of underlying microstructural white matter changes: subtle white matter degeneration, detectable at first only with DTI, may gradually increase before becoming visible as macroscopic WMHs (Pelletier *et al.*, 2015), which in turn give rise to lacunes (Duering *et al.*, 2013). Thus, the difference of microstructural white matter degeneration inside versus outside of WMHs is likely of quantitative rather than qualitative nature, and the same potentially amyloid related pathomechanisms may underlie these white matter alterations.

Primary Alzheimer’s disease pathology may have a direct role in inducing fibre tract degeneration. Converging evidence suggests that amyloid-β and tau-related pathology contribute to chronic hypoperfusion of white matter tissue (Iadecola *et al.*, 1999; Park *et al.*, 2008). Deposition of amyloid-β within microvessels is highly frequent in patients with Alzheimer’s disease dementia (Kalaria, 2016) and associated with reduced blood flow in transgenic mouse models (Han *et al.*, 2008; Saito *et al.*, 2017) and humans (Chung *et al.*, 2009; Dumas *et al.*, 2012). Amyloid-induced vascular constriction, increased vascular resistance, and degeneration of pericytes may further contribute to hypoperfusion (Kisler *et al.*, 2017; Yew *et al.*, 2017). Thus, increased amyloid pathology may contribute to microvascular damage leading to white matter degeneration (for review see Sachdev *et al.*, 2013). It should be noted that although white matter changes were detected subsequent to amyloid and tau pathology in the current study, it is still possible that age-related cerebrovascular pathology may precede primary Alzheimer’s disease pathology (Iturria-Medina *et al.*, 2016) and contribute to white matter changes to a stronger degree in subjects with sporadic Alzheimer’s disease compared to the relatively young subjects with autosomal-dominant Alzheimer’s disease.

Another major open question is the role of microglial activation in the decline of brain integrity in Alzheimer’s disease. Rare partial loss-of-function mutations in the *TREM2* gene are associated with an increase in the risk of Alzheimer’s disease dementia, comparable to that associated with the *APOE* ɛ4 allele (Guerreiro *et al.*, 2013; Jonsson *et al.*, 2013). The protein TREM2 is a receptor primarily expressed in the brain by microglia. TREM2 mutations may be associated with a loss of function affecting microglial chemotaxis, migration, survival, and binding of phospholipids and APOE (Kleinberger *et al.*, 2014, 2017; Atagi *et al.*, 2015; Bailey *et al.*, 2015; Wang *et al.*, 2015; Yeh *et al.*, 2016; Mazaheri *et al.*, 2017; Ulland *et al.*, 2017). TREM2 is actively shed by ADAM10 and ADAM17 (Schlepckow *et al.*, 2017) and released into the CSF (Kleinberger *et al.*, 2014). We have previously shown CSF levels of soluble TREM2 to be increased in the prodromal at-risk state of Alzheimer’s disease (Suarez-Calvet *et al.*, 2016*b*), preceding the estimated age of onset of dementia symptoms by ∼5–10 years as estimated in autosomal-dominant Alzheimer’s disease (Suarez-Calvet *et al.*, 2016*a*). Although it remains largely unclear which ligands bind to the TREM2 receptor, recent *in vivo* experiments in mice suggest that sphingomyelin stemming from damaged myelin (Capodivento *et al.*, 2017) binds to the TREM2 receptor, entailing the activation of microglia (Wang *et al.*, 2015). Thus, in Alzheimer’s disease higher fibre tract damage may result in increased number of microglia and/or higher microglia activity via the TREM2 receptor.

Several caveats should be considered when interpreting the current findings. First, as already mentioned above, the generalizability of the findings from autosomal-dominant Alzheimer’s disease to sporadic late-onset Alzheimer’s disease remains to be tested. Since in autosomal-dominant Alzheimer’s disease, the genetically caused increase in amyloid accumulation may begin already in the third or fourth decade of life, any age-related changes such as vascular pathology that may impact on white matter integrity are unlikely to be detected in autosomal-dominant Alzheimer’s disease, but may still exist in late-onset Alzheimer’s disease.

Second, the current study cannot preclude that white matter alterations may have been due to Wallerian degeneration secondary to grey matter atrophy. However, previous neuroimaging as well as post-mortem brain studies have observed white matter alterations in the presence of minimal or only weak grey matter atrophy (de la Monte, 1989; Zhuang *et al.*, 2012), suggesting that in principle white matter alterations in Alzheimer’s disease are not dependent on grey matter atrophy. Interestingly, a recent longitudinal study in subjects characterized by amyloid and tau PET suggests that fibre tract alterations may be conducive to tau spreading and thus precede grey matter alterations in the projection zones of a tract (Jacobs *et al.*, 2018). The current results encourage future longitudinal studies to assess the role of successive white matter alterations in the progressive expansion of grey matter atrophy.

Third, mean diffusivity was the primary measure to detect white matter alterations in the current study. Mean diffusivity is only one among many possible DTI indices. DTI indices may reflect partially different underlying white matter changes (Alexander *et al.*, 2007), although this is still a matter of intense research (Jones *et al.*, 2013). For exploratory reasons, we also analysed fractional anisotropy, radial diffusivity and axial diffusivity, which showed a substantial spatial overlap particularly in regions including forceps major, forceps minor and posterior parietal part of the cingulum bundle. Consistent with previous studies in mild cognitive impairment (O’Dwyer *et al.*, 2012) and Alzheimer’s disease dementia (Acosta-Cabronero *et al.*, 2010, 2012; Bosch *et al.*, 2012), we observed an increase in axial diffusivity, albeit in a spatially more restricted manner when compared to that of the other DTI indices.

We caution that the behaviour of the DTI indices in the human brain, which is characterized by a high density of crossing fibre that influence DTI measures (Jeurissen *et al.*, 2013), is only partially understood and remains speculative (Jones *et al.*, 2013). DTI alterations may stem from a variety of tissue changes including altered crossing fibres, permeability of the axonal membrane, inflammation, or oedema (Jones *et al.*, 2013). Furthermore, although TBSS is a widely-used approach to analyse DTI, the method has several limitations as previously described, such as susceptibility to misregistration artefacts, crossing fibres, and inaccurate voxel-assignment between neighbouring fibre tracts during the skeletonization procedure (Zalesky, 2011; de Groot *et al.*, 2013; Bach *et al.*, 2014). In particular, long projecting fibres, such as the cortico-spinal tract, cross many other tracts. DTI alterations such as those observed in the corticospinal tract in the current study should be interpreted with caution, as the current TBSS approach cannot be unambiguously attributed to that tract, but potentially are because of alterations in crossing fibres such as the superior longitudinal fasciculus or corpus callosum (Douaud *et al.*, 2011; Teipel *et al.*, 2014; Mito *et al.*, 2018). Also, spatial misregistration may occur particularly in cases of strong mismatch between the template image and the individual images, such as those obtained from elderly subjects with Alzheimer’s disease dementia (Keihaninejad *et al.*, 2012). However, in the current study the first white matter changes were detected in the mutation carriers a decade before symptoms onset, i.e. when no substantial grey matter atrophy is yet present, and at a relatively young age (mean ∼38 years), rendering it unlikely that those DTI alterations were due to gross misregistration artefacts. Furthermore, to reduce the likelihood of partial volume effects, we applied a conservative white matter mask, excluding regions most prone to artefacts such as the fornix. We note that more advanced DTI measures, such as neurite orientation dispersion and density imaging (NODDI) (Zhang *et al.*, 2012), bi-tensor free-water imaging, or fixel-based analysis (Mito *et al.*, 2018) based on multishell-DTI have started to address this problem and will be instrumental in disentangling the different sources of white matter alterations in Alzheimer’s disease.

In conclusion, the current findings suggest that white matter alterations start in circumscribed regions predominantly in posterior parietal and medial prefrontal white matter, preceding the onset of dementia symptoms by more than a decade. These fibre tract changes are an integral part of the Alzheimer’s disease pathological cascade. The current findings encourage future studies to assess the effect of white matter alteration on the cascading breakdown of functional brain networks in Alzheimer’s disease.

## Supplementary Material

Supplementary Data

## Notes

See Jacobs and Buckley (doi:10.1093/brain/awy243) for a scientific commentary on this article.

## Abbreviations

DTI: diffusion tensor imaging
EYO: estimated years from symptom onset
PIB: Pittsburgh compound B
PSMD: peak-width of skeletonized mean diffusivity
TBSS: tract-based spatial statistics
WMH: white matter hyperintensity
