# Supplementary material for: White matter diffusion alterations precede symptom onset in autosomal dominant Alzheimer’s disease
Source: Brain. 2018 Sep 25;141(10):3065–80. doi: 10.1093/brain/awy229 (PMC6158739; doi:10.1093/brain/awy229)
Supplement: Supplementary Data [file awy229_supplementary_material.pdf]

SUPPLEMENTARY MATERIAL

Supplemenatary methods 1:

WMH Segmentation

Briefly, the FLAIR images were bias-corrected and segmented into the three tissue segments (white matter, GM and CSF) using the FAST toolbox of FSL. The resulting CSF segment contained a mixture of voxels with high intensity values (stemming from WMH) and low intensity values (stemming from CSF). In a second step, the WMH and CSF segments were segregated by histogram segmentation (Otsu method (Otsu, 1979)). The resulting WMH maps underwent manual editing to remove any potential artifacts. To this end, two independent raters (MÁAC, MS-C) edited each subject's WMH map by classifying each voxel cluster as either a WMH or an artefact. This was done with an in-house GUI developed in MATLAB (MathWorks, Natick, MA, USA). The inter-rater reliability analysis of the WMH classification resulted in a mean Dice coefficient of 0.81. Only voxels considered as WMH by both raters were included for the analysis.

For each subject, the T1 scans were segmented into grey-matter (GM), white-matter (white matter) and cerebrospinal fluid (CSF) tissue segments using SPM12 (http://www.fil.ion.ucl.ac.uk/spm/). Individual brain masks were generated by combining and binarizing these tissue segments, and subsequently used for brain extraction of the T1. High dimensional spatial normalization parameters for registration to the standard MNI space were estimated,

In order to normalize the resulting WMH maps to MNI space, spatial normalization parameters were estimated based on the grey matter segment (derived from T1 MRI scans), using the DARTEL toolbox of SPM12 (Ashburner, 2007). The brain-extracted FLAIR scan for each subject was linearly registered to the subject's T1 scan, applying co-registration to

the WMH maps. The registered WMH maps were subsequently normalized to MNI using the nonlinear registration parameters derived by the DARTEL procedure in a previous step.

In order to derive tract-specific WMH, we superimposed the normalized WMH map onto the Johns Hopkins University (Johns-Hopkins-University) white-matter tractography atlas (Hua *et al.*, 2008) (thresholded at 25%). The intersection between the binary WMH map and each tract was defined as the tract-specific WMH, and the remaining part of the tract as normal appearing white matter.

Supplemenatary methods 2:

Determination of GM volume in projection zones of fiber tracts

The fiber tract of interest (i.e. those tracts that showed significant MD changes in the mutation carriers) were reconstructed based on the Johns-Hopkins-University-DTI-whole-brain-tractography template as implemented in ExploreDTI (Wakana *et al.*, 2007). To this end, we placed pairs of predefined ROIs at anatomical landmarks according to a previously established protocol (Wakana *et al.*, 2007) to determine those fiber tracts that pass through the ROIs. Thus, each tract of interest (forceps major, forceps minor, inferior fronto-occipital fasciculus, inferior longitudinal fasciculus, superior longitudinal fasciculus, cortico-spinal tract, and hippocampal cingulum bundle) were reconstructed within the Johns-Hopkins-University DTI template in MNI space. In order to determine the grey matter projection zone, the binarized MNI152 grey matter template (> 0.3) was superimposed onto the DTI template containing the reconstructed tracts.

Next, for each of these tracts, the grey matter projection zone was determined as follows (see Supplementary figure 1 for illustration of the different steps). First, the trajectories of the streamlines that comprised each fiber tract was determined. Specifically, for a given streamline, we computed the tangents at 1-mm intervals across the 5-mm long terminal

sections of that streamline (Supplementary figure 1A; only two tangents shown for simplicity). For each streamline, the resulting 5 derivatives (tangents), were averaged in order to determine the average direction of streamline projection. Each streamline was projected along the estimated direction into the grey matter, as defined by the grey matter mask superimposed onto the fiber tract template in MNI space (Supplementary Figure 1B). The propagation of the streamline was continued throughout the grey matter until it reached the outer surface of the cortex. The projection zone for each streamline was defined as the 3-voxel margin along the projected streamline within the grey matter mask, and the total projection zone for each fiber tract was the sum of all the streamline's projection zones for that tract. As an example, a 2D axial view of the projection zone estimated for the forceps minor is shown Supplementary Figure 1C (blue area), where the grey matter projection zone was delineated after masking with the binary grey matter mask in MNI space (Supplmentary figure 1D, red area). Finally, for each tract, the thus generated grey-matter projection zone was superimposed onto each subject's spatially normalized grey matter map in MNI space (for spatial normalization see Methods). The GM volume of a tract's projection zone was computed as the sum of voxel values of the normalized and modulated grey matter within the projection-zone mask (Mak *et al.*, 2011).

Supplementary results

Mapping of group differences in secondary DTI indices across EYO

We found significant interactions of EYO by mutation status on fractional anisotropy (Supplementary Fig. 2, Supplementary Table 3), radial diffusivity (Supplementary Fig. 3, Supplementary Table 4) and axial diffusivity (Supplementary Fig. 4, Supplementary Fig. 5). For fractional anisotropy, the interactions concentrated in regions similar to the MD changes, but were not as extended. For radial diffusivity, the spatial extent of the significant

interactions was similar to MD, but the peaks clustered in portions of the superior longitudinal fasciculi. For axial diffusivity, however, the interactions were more sparsely distributed than for any of the other indexes, but also found mainly in posterior and parietal regions of the white matter.

Supplementary Fig. 5 top shows the spatial overlap of the interaction effects (EYO x mutation status) for MD, fractional anisotropy, and radial diffusivity. Visual inspection shows a substantial overlap between the statistical maps, particularly in the posterior parietal lobe. When adding the statistical map of significant interaction effects for axial diffusivity (Supplementary Fig. 5 bottom), the overlap between all maps is reduced due to the sparse distribution of significant interaction effects for axial diffusivity.

SUPPLEMENTARY FIGURES

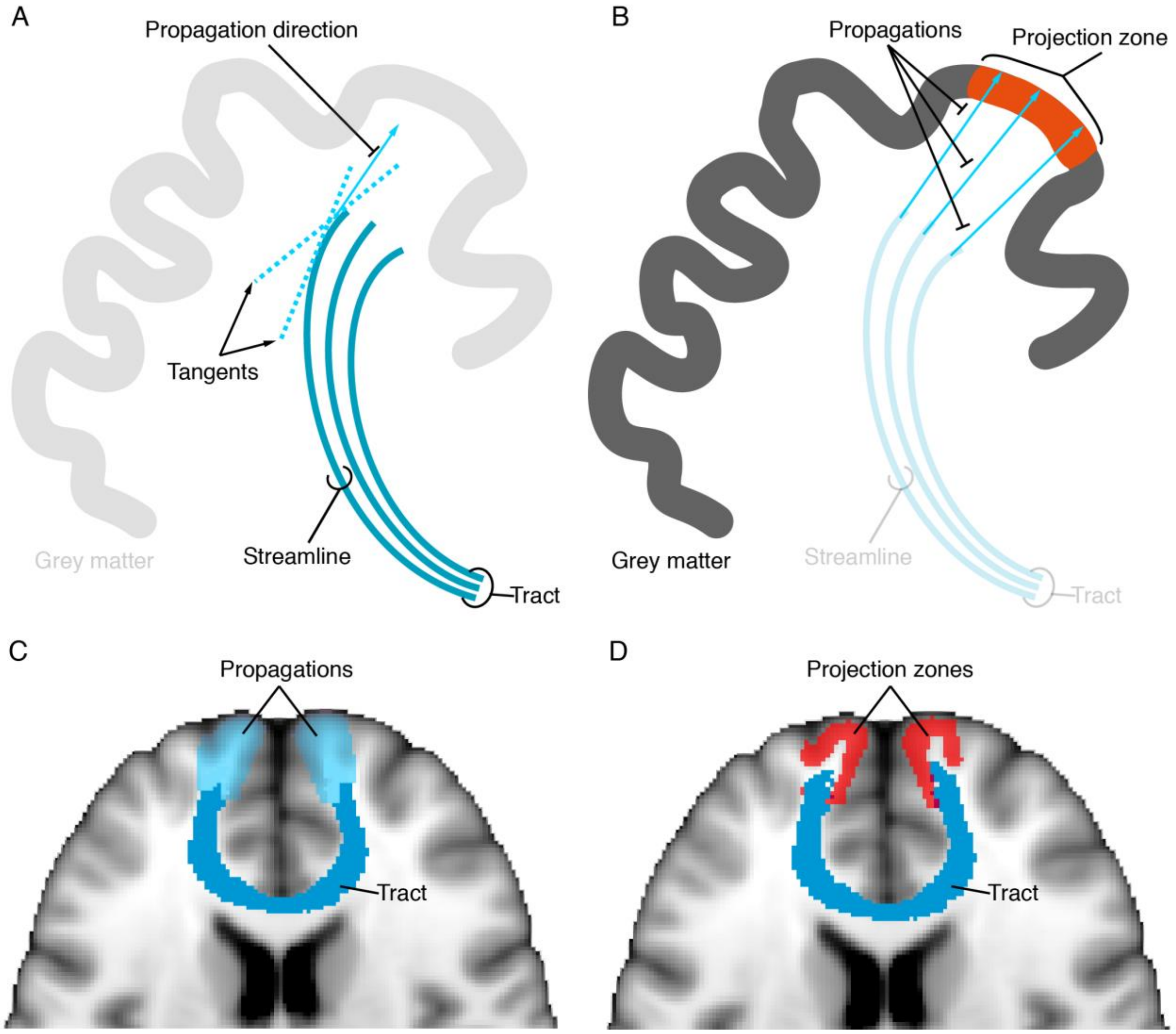

Supplementary Figure 1: Schematic illustration of the different steps to estimate a tract's projection zone illustrated here for the forceps minor (for details see Supplementary Methods 2).

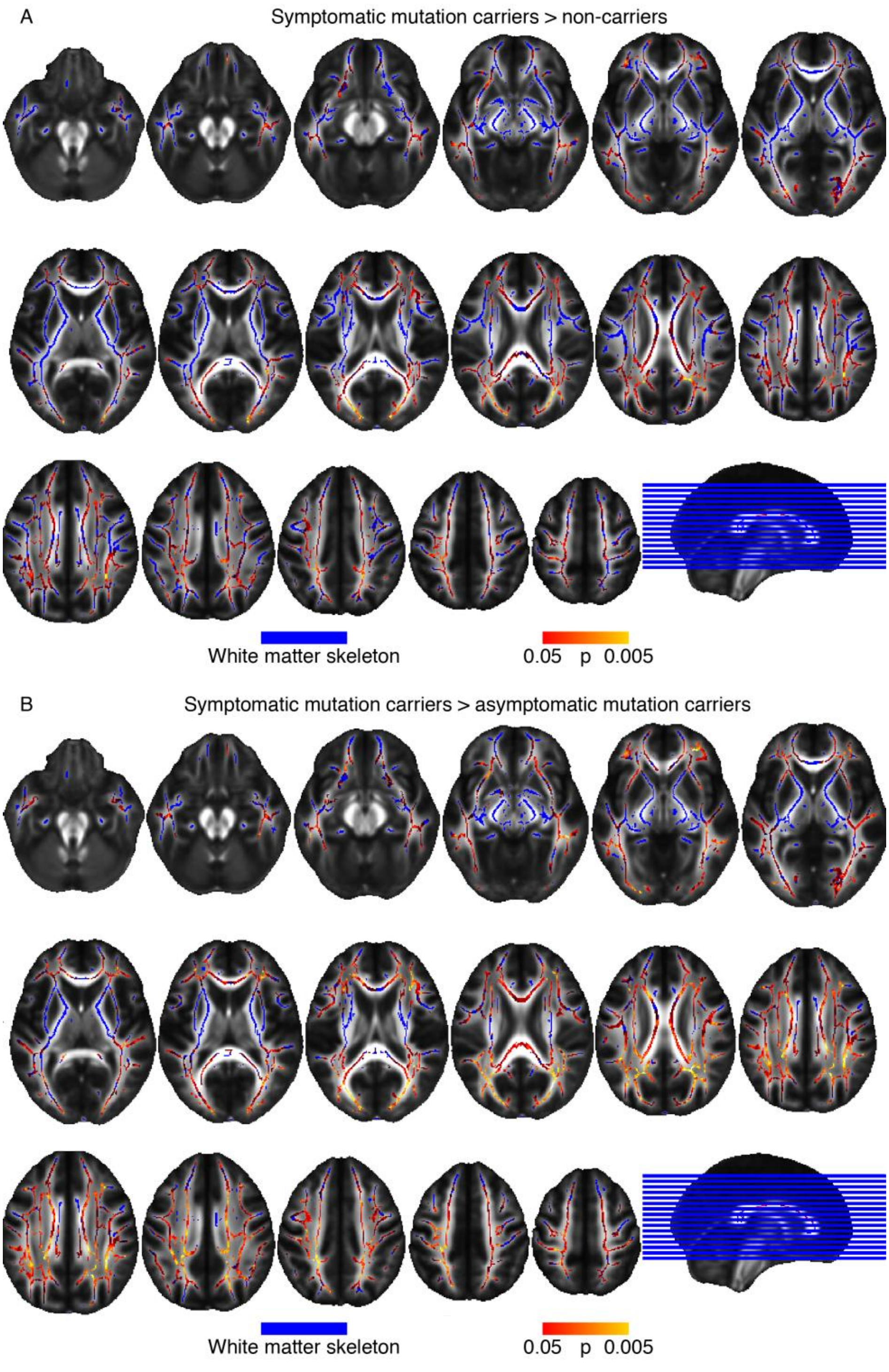

Supplementary Figure 2: (A) differences in MD between non-carriers and symptomatic carriers (B) differences in MD between symptomatic and asymptomatic carriers.

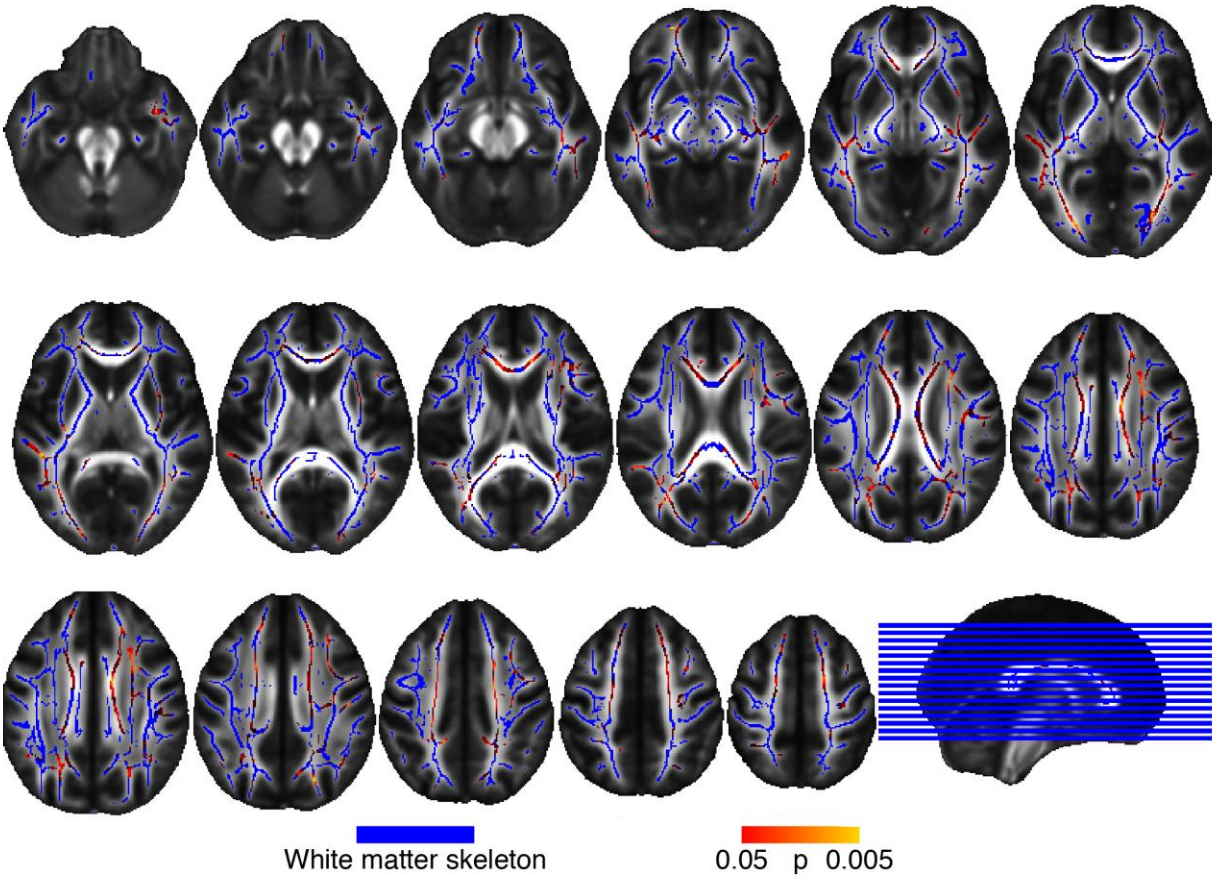

Supplementary Figure 3: Effect of the interaction EYO x Mutation status on fractional anisotropy (red-yellow) superimposed on the white matter skeleton (blue), FWE-corrected at p = 0.05.

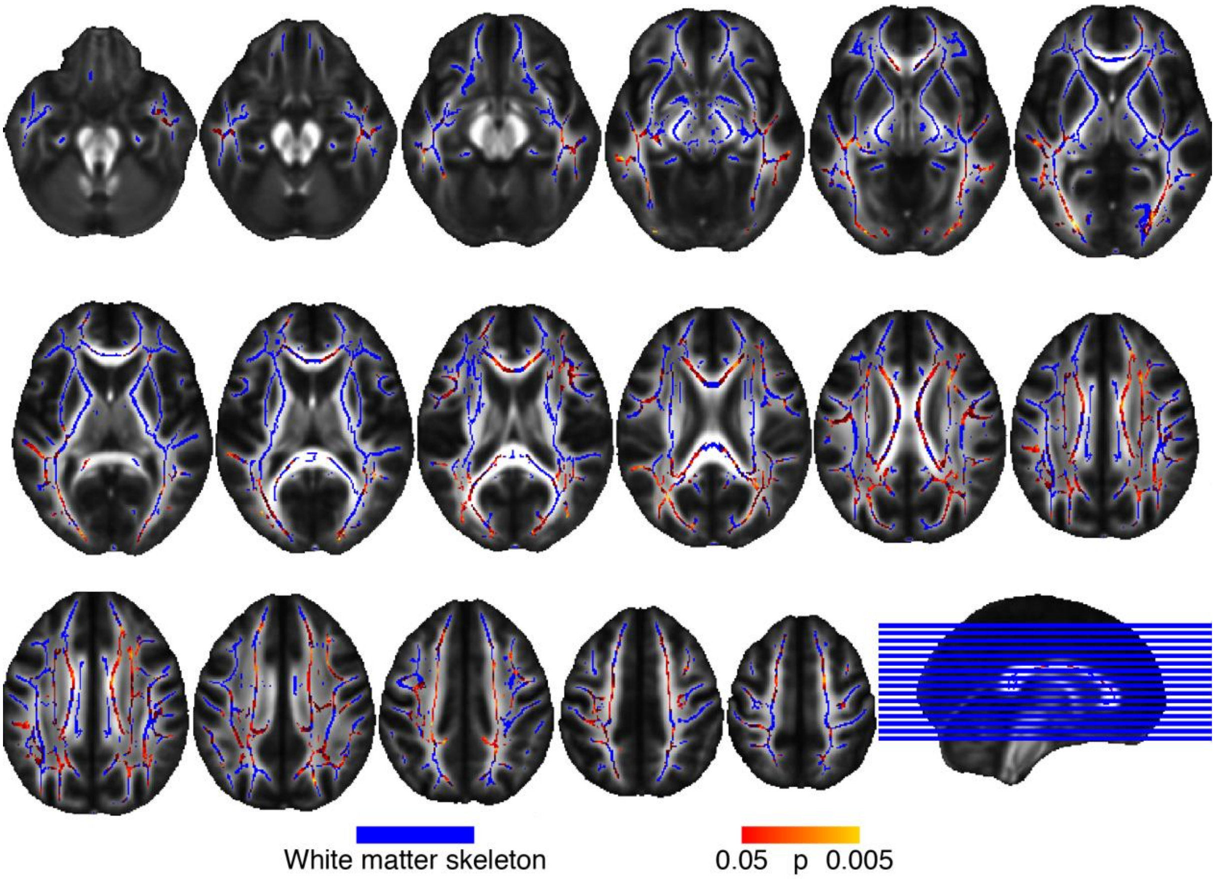

Supplementary Figure 4: Effect of the interaction EYO x Mutation status on radial diffusivity (red-yellow) superimposed on the white matter skeleton (blue), FWE-corrected at p = 0.05.

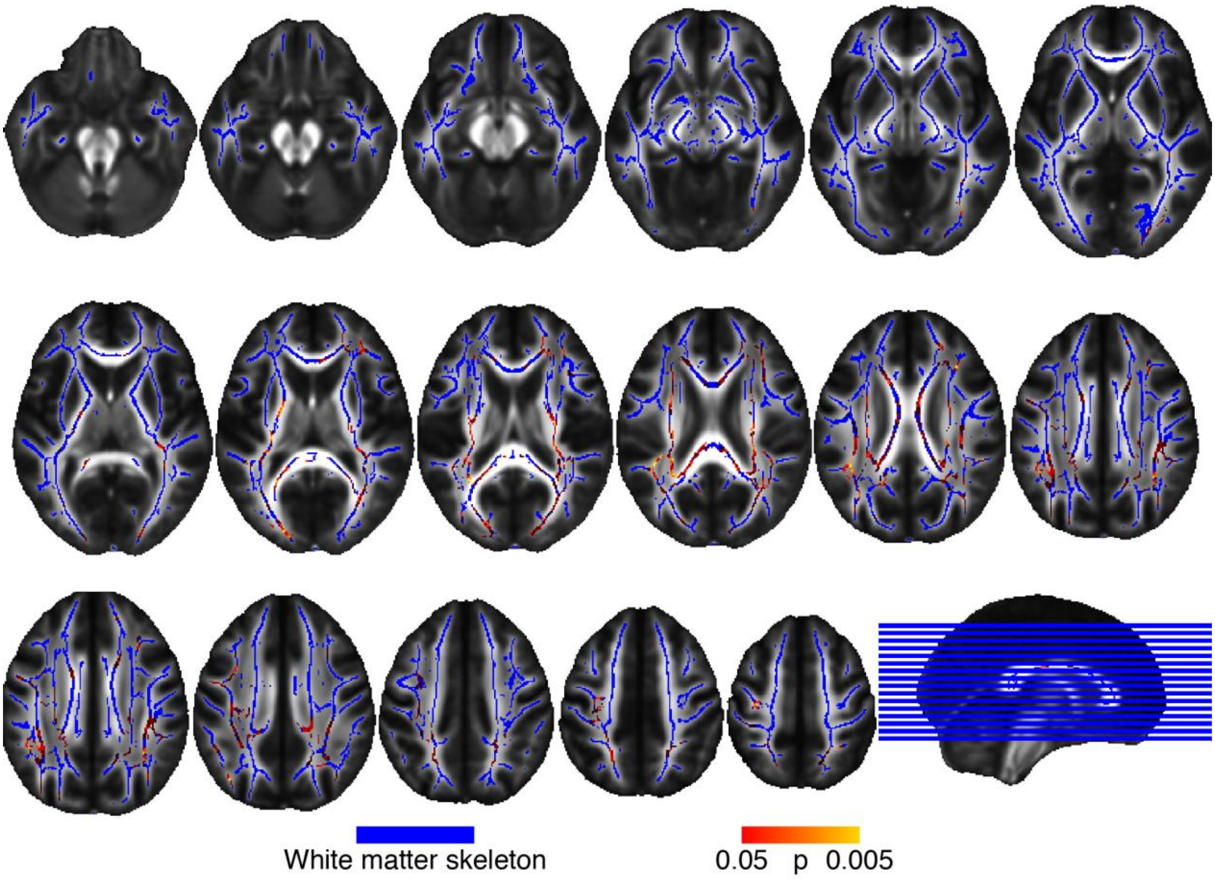

Supplementary Figure 5: Effect of the interaction EYO x Mutation status on axial diffusivity (red-yellow) superimposed on the white matter skeleton (blue), FWE-corrected at p = 0.05.

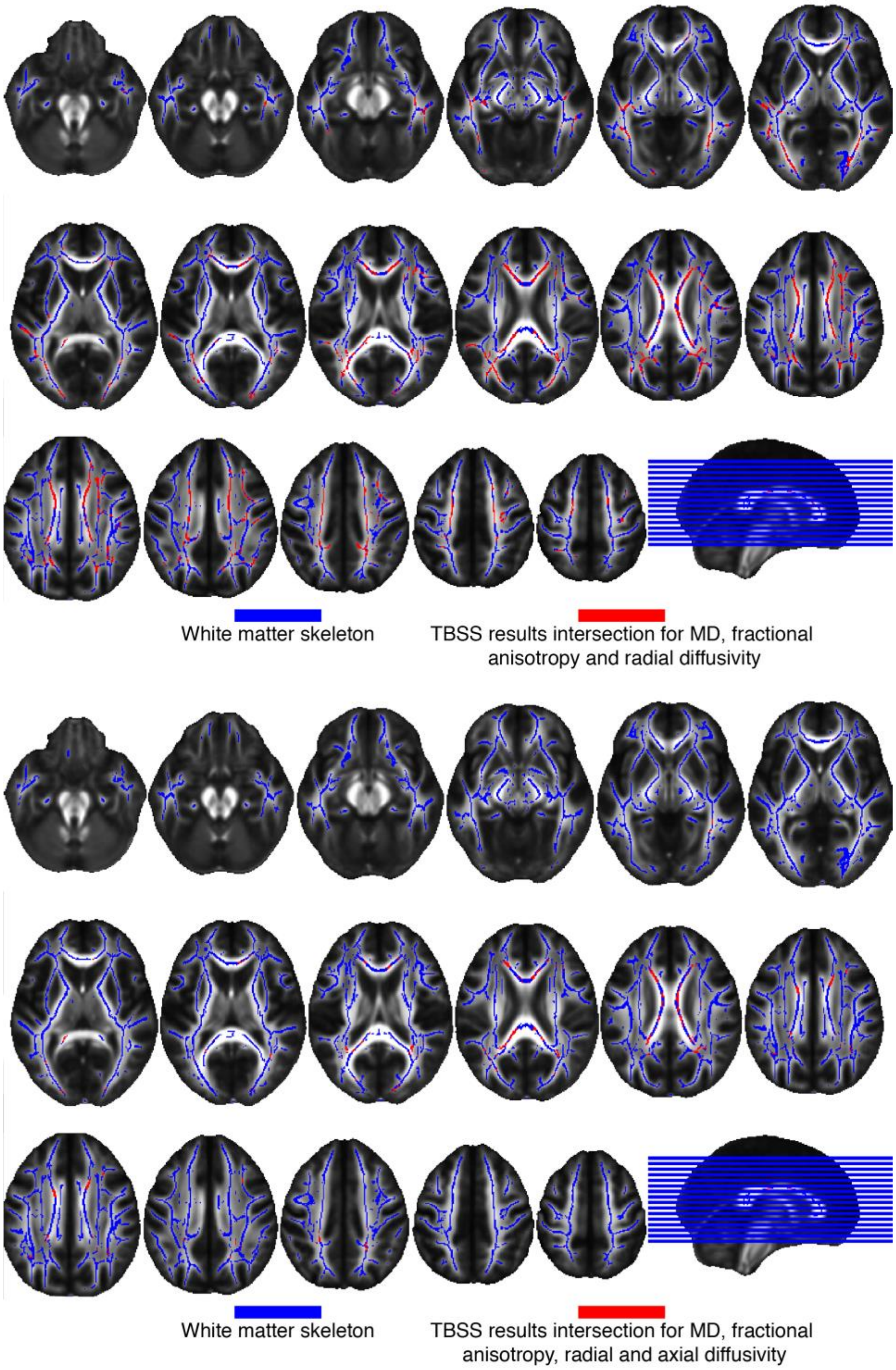

Supplementary Figure 6: (A) Intersection of the TBSS results for MD, fractional anisotropy and radial diffusivity. (B) Intersection of TBSS results for all DTI indices.

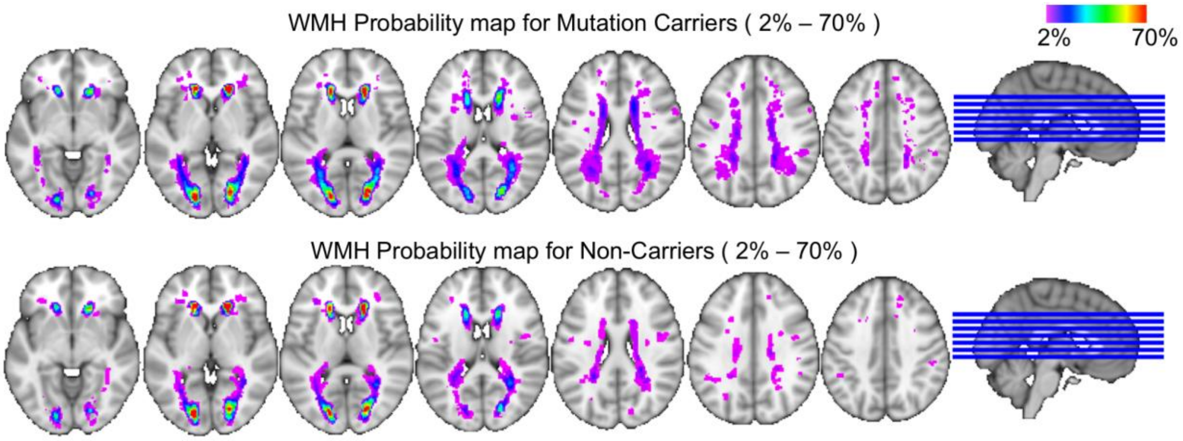

Supplementary Figure 7: WMH probability maps in the carriers (top) and non-carriers (bottom).

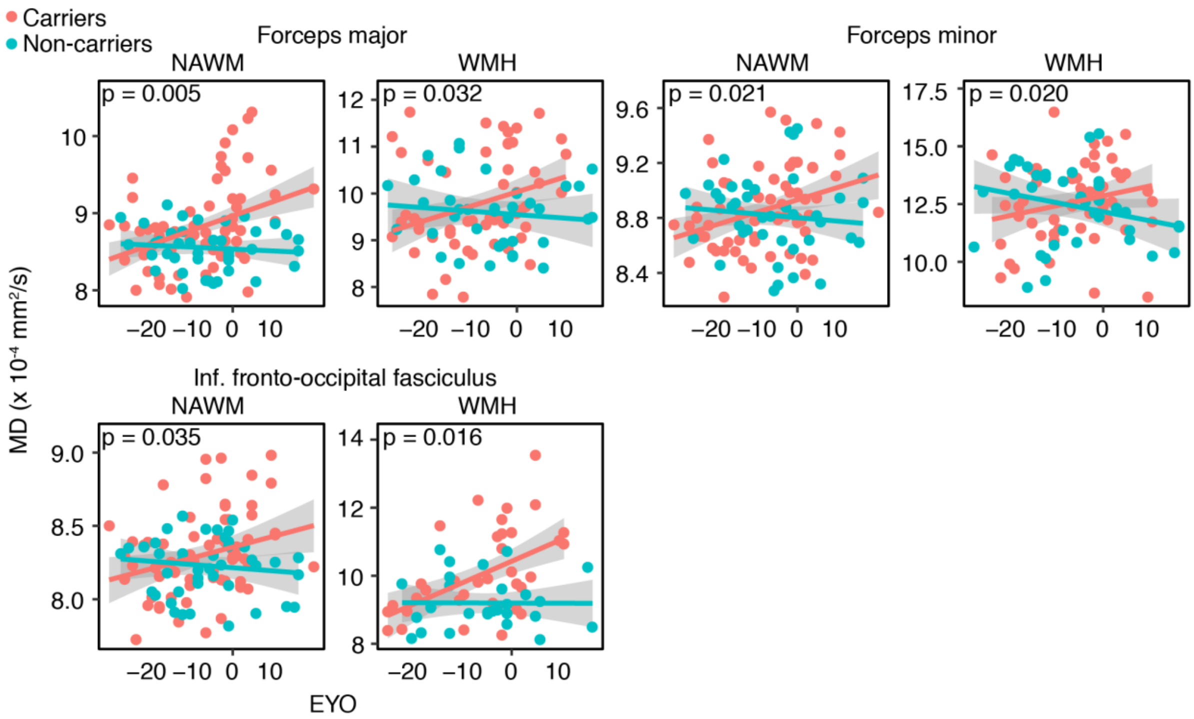

Supplementary Figure 8: MD as a function of EYO for carriers (red) and non-carriers (cyan) within WMH and normal appearing white matter. Regression lines and 95% CI (shaded bands) are shown. Tracts with low proportion of participants showing WMH (< 50%) were omitted from this analysis.

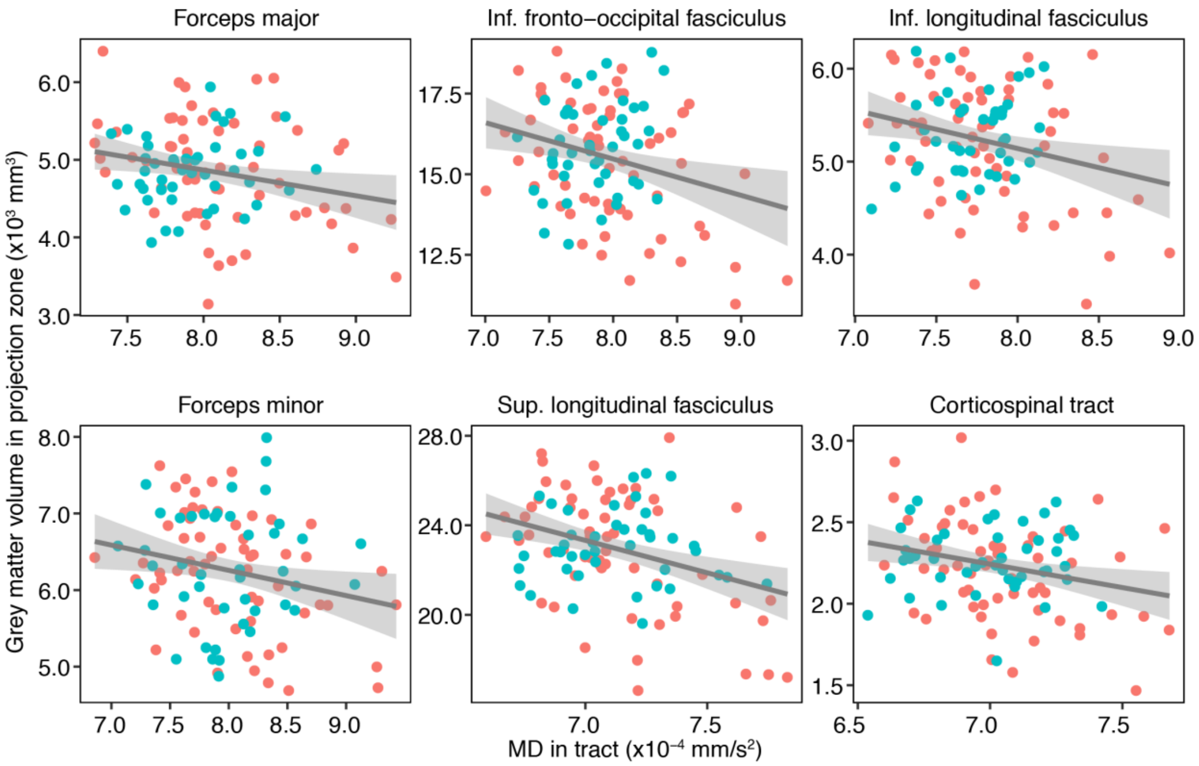

Supplementary Figure 9: GM volume in projection zones as a function of MD within the tract for all 6 significant tracts. Forceps major: B(SE) = –0.27(0.09), p(FDR) = 0.006; inferior fronto-occipital fasciculus: B(SE) = –0.32(0.10), p(FDR) = 0.004; inferior longitudinal fasciculus: B(SE) = –0.29(0.10), p(FDR) = 0.006; forceps minor: B(SE) = –0.25 (0.10), p(FDR) = 0.014; superior longitudinal fasciculus: B(SE) = –0.33(0.10), p(FDR) = 0.004; corticospinal tract: B(SE) = –0.24(0.09), p(FDR) = 0.017. FDR correction computed over all 7 tracts, only 6 significant tracts shown (hippocampal cingulum bundle was not significant even before FDR correction). We used a permutation test to assess whether the associations were region-specific. Briefly, we estimated for each of the six tracts, the association between tract-based MD and the grey matter volumes of each of the 6 projection zones and rank-ordered the regression coefficients. We found that for each tract, the association between the tract-based MD and grey matter volume in the anatomically correct projection area ranked highest or second. The likelihood for such a result pattern by chance was $p < 0.001$.

SUPPLEMENTARY TABLES

Supplementary Table 1: Location and MNI coordinates of peak T-statistic values of significant clusters for MD ~ symptomatic carriers > non-carriers.

Cluster	Tract	Reference	X	Y	Z	T-value
1	Forceps major	Occipital lobe	–10	–88	18	5.21
2	Anterior thalamic radiation R	Frontal lobe	33	24	31	3.93
3	Cingulum	Frontal lobe	–13	17	26	4.23
4	Inferior fronto-occipital fasciculus R	Temporal lobe	40	–30	–15	4.32
5	Corticospinal tract R	Parietal lobe	19	–36	63	4.27
6	Superior longitudinal fasciculus R	Frontal lobe	19	16	38	3.52

Legend: R = Right, L = Left. Notes: 8 clusters with volume > 5 x 5 x 5 mm^3 were found. Tracts are taken from the Johns-Hopkins-University White-Matter Tractography Atlas. For orientation, closest cortical regions are taken from the MNI Structural Atlas. Regions/Tracts with peak probability are shown. X, Y and Z coordinates in mm.

Supplementary Table 2: Location and MNI coordinates of peak T-statistic values of significant clusters for MD ~ symptomatic carriers > asymptomatic carriers.

**Cluster**	**Tract**	**Reference**	**X**	**Y**	**Z**	**T-value**
1	Inferior fronto-occipital fasciculus R	Parietal lobe	29	–67	22	6.72
2	Inferior longitudinal fasciculus L	Temporal lobe	–43	–44	–4	4.72
3	Uncinate L	Frontal lobe	–21	22	–10	4.34
4	Anterior thalamic radiation L	Frontal lobe	–28	40	15	4.28
5	Superior longitudinal fasciculus L	Temporal lobe	–52	–33	8	3.80
6	Inferior fronto-occipital fasciculus R	Frontal lobe	23	24	–5	3.78
7	Forceps minor	Frontal lobe	–20	46	15	3.97
8	Corticospinal tract R	Parietal lobe	20	–12	43	4.67

Legend: R = Right, L = Left. Notes: 8 clusters with volume > 5 x 5 x 5 mm^3 were found. Tracts are taken from the Johns-Hopkins-University White-Matter Tractography Atlas. For orientation, closest cortical regions are taken from the MNI Structural Atlas. Regions/Tracts with peak probability are shown. X, Y and Z coordinates in mm.

Supplementary Table 3: Location and MNI coordinates of peak T-statistic values of significant clusters for fractional anisotropy ~ Mutation x EYO

**Cluster**	**Tract**	**Reference**	**X**	**Y**	**Z**	**T-value**
1	Cingulum R	Frontal lobe	13	–3	33	4.28
2	Forceps major	Occipital lobe	–22	–82	1	4.37
3	Superior longitudinal fasciculus R	Frontal lobe	26	12	29	4.23
4	Superior longitudinal fasciculus L	Temporal lobe	–45	–35	6	5.11
5	Inferior fronto-occipital fasciculus R	Occipital lobe	27	–74	2	3.77
6	Superior longitudinal fasciculus L	Temporal lobe	57	–31	–8	3.79
7	Inferior longitudinal fasciculus R	Temporal lobe	49	–7	–10	3.53
8	Superior longitudinal fasciculus R	Parietal lobe	39	–56	34	3.76

Legend: R = Right, L = Left. Notes: 8 clusters with volume > 5 x 5 x 5 mm^3 were found. Tracts are taken from the Johns-Hopkins-University White-Matter Tractography Atlas. For orientation, closest cortical regions are taken from the MNI Structural Atlas. Regions/Tracts with peak probability are shown. X, Y and Z coordinates in mm.

Supplementary Table 4: Location and MNI coordinates of peak T-statistic values of significant clusters for radial diffusivity ~ Mutation x EYO

**Cluster**	**Tract**	**Reference**	**X**	**Y**	**Z**	**T-value**
1	Forceps major	Occipital lobe	–24	–79	1	4.71
2	Superior longitudinal fasciculus R	Frontal lobe	30	11	35	3.98
3	Superior longitudinal fasciculus L	Temporal lobe	–45	–35	6	4.22
4	Superior longitudinal fasciculus L	Frontal lobe	–39	–1	23	3.23
5	Superior longitudinal fasciculus L	Parietal lobe	–23	–11	45	3.47
6	Uncinate fasciculus L	Frontal lobe	–14	26	44	3.45

Legend: R = Right, L = Left. Notes: 8 clusters with volume > 5 x 5 x 5 mm^3 were found. Tracts are taken from the Johns-Hopkins-University White-Matter Tractography Atlas. For orientation, closest cortical regions are taken from the MNI Structural Atlas. Regions/Tracts with peak probability are shown. X, Y and Z coordinates in mm.

Supplementary Table 5: Location and MNI coordinates of peak T-statistic values of significant clusters for axial diffusivity ~ Mutation x EYO

**Cluster**	**Tract**	**Reference**	**X**	**Y**	**Z**	**T-value**
1	Superior longitudinal fasciculus L	Parietal lobe	–38	–42	25	4.44
2	Forceps major	Occipital lobe	–26	–52	18	4.86
3	Corticospinal tract R	Parietal lobe	27	–29	24	3.89
4	Forceps major	Occipital lobe	–19	–87	12	4.30
5	Superior longitudinal fasciculus L	Frontal lobe	–27	2	22	2.98
6	Forceps major	Parietal lobe	–17	–45	10	3.59
7	Anterior thalamic radiation R	Parietal lobe	–5	–27	23	3.10

Legend: R = Right, L = Left. Notes: 8 clusters with volume > 5 x 5 x 5 mm^3 were found. Tracts are taken from the Johns-Hopkins-University White-Matter Tractography Atlas. For orientation, closest cortical regions are taken from the MNI Structural Atlas. Regions/Tracts with peak probability are shown. X, Y and Z coordinates in mm.

Supplementary Table 6: Location and MNI coordinates of peak T-statistic values of significant clusters for MD ~ CSF sTREM2 in carriers (Figure 6A).

**Cluster**	**Tract**	**Reference**	**X**	**Y**	**Z**	**T-value**
1	Cingulum L	Frontal lobe	–14	13	28	4.93
2	Inferior longitudinal fasciculus R	Temporal lobe	39	–47	–4	4.27
3	Inferior fronto-occipital fasciculus R	Frontal lobe	34	28	15	3.88
4	Inferior longitudinal fasciculus L	Temporal lobe	–44	–17	–17	4.53
5	Corticospinal tract R	Parietal lobe	25	–29	30	3.62
6	Uncinate fasciculus L	Temporal lobe	–35	–3	–14	3.84
7	Superior longitudinal fasciculus R	Parietal lobe	52	–39	31	3.34

Legend: R = Right, L = Left. Notes: 8 clusters with volume > 5 x 5 x 5 mm^3 were found. Tracts are taken from the Johns-Hopkins-University White-Matter Tractography Atlas. For orientation, closest cortical regions are taken from the MNI Structural Atlas. Regions/Tracts with peak probability are shown. X, Y and Z coordinates in mm.

Supplementary Table 7: Location and MNI coordinates of peak T-statistic values of significant clusters for MD ~ CSF A$\beta_{1\text{-}42}$ x total tau in carriers (Figure 6B).

**Cluster**	**Tract**	**Reference**	**X**	**Y**	**Z**	**T-value**
1	Superior longitudinal fasciculus R	Parietal lobe	–53	–46	21	5.75
2	Superior longitudinal fasciculus R	Parietal lobe	49	–20	30	5.92
3	Inferior longitudinal fasciculus R	Temporal lobe	51	–16	1	4.51
4	Corticospinal tract R	Parietal lobe	25	–10	17	4.99
5	Inferior longitudinal fasciculus R	Temporal lobe	36	–56	–8	3.55
6	Cingulum	Parietal lobe	–15	–31	29	3.71
7	Anterior thalamic radiation L	Frontal lobe	–31	38	11	4.56
8	Uncinate R	Frontal lobe	29	30	–7	4.57

Legend: R = Right, L = Left. Notes: 8 clusters with volume > 5 x 5 x 5 mm^3 were found. Tracts are taken from the Johns-Hopkins-University White-Matter Tractography Atlas. For orientation, closest cortical regions are taken from the MNI Structural Atlas. Regions/Tracts with peak probability are shown. X, Y and Z coordinates in mm.

Supplementary Table 8: Volume and anatomical labels of the brain regions in the fiber tracts' projection zone

**Tract**	**Volume (mm^3)**	**AAL regions in the projection zone**
Forceps major	10962	Cuneus
		Superior occipital gyrus
		Calcarine fissure cortex
Inf. fronto-occipital fasciculus	31124	Inferior frontal gyrus (orbital)
		Middle occipital gyrus
		Middle frontal gyrus (orbital)
		Calcarine fissure cortex
		Middle frontal gyrus
Forceps minor	14076	Superior frontal gyrus (medial)
		Superior frontal gyrus
		Middle frontal gyrus (orbital)
		Superior frontal gyrus (orbital)
		Gyrus rectus
Inf. longitudinal fasciculus	8655	Calcarine fissure cortex
		Temporal pole (middle temporal gyrus)
		Lingual gyrus
		Middle occipital gyrus
		Inferior occipital gyrus
Corticospinal tract	7718	Postcentral gyrus
		Paracentral lobule
		Precentral gyrus
		Superior parietal gyrus
Sup. longitudinal fasciculus	39698	Middle temporal gyrus

		Inferior temporal gyrus
		Inferior frontal gyrus (opercular)
		Precentral gyrus
		Inferior frontal gyrus (triangular)
Hippocampal cingulum	39582	Calcarine fissure cortex
		Cuneus
		Precuneus
		Temporal pole (middle temporal gyrus)
		Parahippocampal gyrus

Legend: AAL = Automatic Anatomical Labeling ROIs (Tzourio-Mazoyer *et al.*, 2002).
